# Sex-specific effects of resveratrol and metformin on mitochondrial function and inflammatory activation in a TNF-α-driven human iPSC-derived endothelial dysfunction model

**DOI:** 10.3389/fimmu.2026.1903774

**Published:** 2026-08-13

**Authors:** Julia Temp, Elisabeth Tamara Strässler, Julia Freitag, Haiyan Wu, Natalie Haritonow, Ulf Landmesser, Sofya Podzniakova, Maria Luisa Barcena

**Affiliations:** 1 Department of Urology, Eberhard Karls University of Tuebingen, Tuebingen, Germany; 2 Department of Cardiology, Angiology and Intensive Care Medicine, Deutsches Herzzentrum der Charité, Berlin, Germany; 3 Partner Site Berlin, German Centre for Cardiovascular Research, Berlin, Germany; 4 Department of Geriatrics and Medical Gerontology, Charité Universitätsmedizin Berlin, Freie Universität Berlin, Humboldt-Universität zu Berlin, Berlin Institute of Health, Berlin, Germany; 5 Department of Respiratory, Zheijan Hospital, Zhejiang, Hangzhou, China; 6 Berlin Institute of Health at Charité, Berlin, Germany; 7 Friede Springer - Cardiovascular Prevention Center @Charité, Charite Universitätsmedizin Berlin, Berlin, Germany; 8 Climate and Health Program, Barcelona Institute for Global Health, Barcelona, Spain

**Keywords:** immunometabolic responses, inflammatory dysfunction model, iPSC-EC, metformin, resveratrol, sex differences

## Abstract

Endothelial dysfunction and mitochondrial impairment are key early events in atherogenesis and exhibit sex-specific patterns. Resveratrol and metformin are cardiometabolic modulators with antioxidative and mitochondrial effects, but their sex-dependent actions in human iPSC-derived endothelial cells (iPSC-EC) remain unclear. iPSC-EC generated from peripheral blood mononuclear cells of six male and six female donors were stimulated with TNF-α to induce a pro-atherogenic phenotype and subsequently treated with resveratrol or metformin. Mitochondrial ROS production, mitochondrial content, endothelial permeability, mitochondrial respiration, inflammatory mediators and LDH release were quantified in a sex-stratified manner. In this TNF-α-driven human iPSC-EC inflammatory dysfunction model, male iPSC-EC displayed stronger mitochondrial superoxide signal, inflammatory activation and endothelial barrier disruption, whereas female iPSC-EC showed a trend towards impaired mitochondrial respiration and reduced IL-10. Resveratrol and metformin trend towards reduced selected TNF-α associated endothelial injury markers. Resveratrol and metformin attenuated TNF-α–associated oxidative stress, cytokine secretion, permeability and LDH release, with male cells mainly benefiting from decreased mitochondrial superoxide signal and inflammation, and female cells showing non-significant tendencies towards improved mitochondrial respiration and restoration of IL-10. These findings highlight cellular sex as a critical determinant of endothelial immunometabolic responses to inflammatory stress and suggest that resveratrol- and metformin-based strategies for vascular protection should be evaluated in a sex-aware manner.

## 1 Introduction

Cardiovascular diseases remain the leading cause of death worldwide and are strongly driven by atherosclerosis, a chronic inflammatory disease of the arterial wall (1, 2). Men typically develop atherosclerotic complications earlier and more frequently than women (3, 4), whereas women show a steeper risk increase after menopause (5), highlighting the importance of both age and biological sex for vascular disease susceptibility. Endothelial cells are central orchestrators of early atherogenesis (6, 7); exposure to pro-atherogenic stimuli such as oxidized LDL, disturbed shear stress, hyperglycemia, or pro-inflammatory cytokines (e.g., TNF-α) promote oxidative stress, endothelial activation, increased permeability, and expression of adhesion molecules, thereby facilitating leukocyte recruitment and plaque initiation (8, 9).

We and others have recently shown that endothelial responses to inflammatory stress are intrinsically sex-dependent, even in the absence of systemic sex hormones (10–12). In human umbilical vein endothelial cells (HUVEC) from male and female donors, TNF-α and pro-inflammatory monocyte supernatants enhanced adhesion molecule expression, cytokine secretion and mitochondrial ROS formation, with a more pronounced pro-atherogenic and pro-oxidative phenotype in male cells (11). Likewise, in human iPSC-derived endothelial cells (iPSC-EC), TNF-α increased reactive oxygen species (ROS) generation, permeability, VCAM-1 expression, caspase-1 activity and IL-6 release preferentially in male cells (13), suggesting that cellular sex is a critical determinant of endothelial vulnerability to inflammatory injury (10, 12). These findings are consistent with broader evidence indicating sex differences in mitochondrial function, autophagy and cellular senescence in cardiovascular aging (14–16).

Resveratrol, a polyphenolic Sirt1 activator, has been widely studied as a cardioprotective compound with antioxidative, anti-inflammatory and beneficial mitochondrial effects (17–20). In experimental models, resveratrol improves endothelial function, reduces ROS formation, enhances mitochondrial biogenesis (21, 22). Metformin, another caloric-restriction mimetic, similarly activates AMPK, improves mitochondrial function and dampens inflammatory responses in vascular and cardiac cells (23, 24). However, potential sex-specific differences in the endothelial and mitochondrial response to resveratrol or metformin remain poorly understood, and human data in iPSC-derived endothelial models that preserve donor-specific genetic and epigenetic backgrounds are scarce.

iPSC-EC offer a powerful platform to dissect sex-specific responses to inflammatory and metabolic stress under standardized conditions, independent of confounding by circulating hormones, comorbidities and medications (18, 25). Building on our previous work in HUVEC (11), we here established a TNF-α–driven *in vitro* inflammatory dysfunction model in male and female iPSC-EC to systematically assess the effects of resveratrol compared with metformin on mitochondrial function, ROS generation, endothelial barrier integrity and inflammatory activation.

We hypothesized that (i) TNF-α would induce a more pronounced oxidative and inflammatory endothelial phenotype in male than in female iPSC-EC, (ii) resveratrol and metformin would exert overlapping but not identical protective effects on mitochondrial respiration and endothelial function, and (iii) these protective effects would exhibit sex-specific patterns, with resveratrol particularly improving mitochondrial function in female iPSC-EC and reducing oxidative damage and inflammatory activation in male cells.

## 2 Material and methods

### 2.1 Cell culture

Human iPSC generated from peripheral blood mononuclear cells (PBMC) obtained from six male and six female donors were kindly provided by the laboratory of Prof. Joost Sluijter at University Medical Centre Utrecht, The Netherlands. The iPSCs were differentiated into endothelial cells according to the protocol published by Patsch and colleagues (26). In short, endoderm induction was performed via WNT activation through stimulation with 6µM CHIR-99021 and 50 ng/ml BMP-4 in N2B27 medium [DMEM/F12:Neurobasal medium 1:1 with 2% B27 Supplement minus Vitamin A and 1% N-2 Supplement (ThermoFisher, Germany)] for 72 h, followed by endothelial differentiation in StemPro-34 medium (ThermoFisher, Germany) supplemented with 200 ng/ml VEGF-A (PeproTech, Germany) and 2 µM Forskolin (PeproTech, Germany) for 48 h. Endothelial cells were purified with CD144 microbeads (Miltenyi, Germany) and cultured in endothelial cell growth medium 2 (Promo Cell GmbH, Heidelberg, Germany) in a humidified incubator at 37°C and 5% CO_2_. After purification, over 99% of iPSC-ECs were CD31+ and over 95% were CD309+ as assessed via flow cytometry. For experiments, iPSC-ECs between passage 3–5 were used. The medium was completely replaced every 2 days to ensure optimal nutrient supply and removal of metabolic waste.

Cells were harvested for passaging by enzymatic dissociation with trypsin/EDTA (PAN Biotech, Aidenbach, Germany) and resuspended in fresh Endothelial Cell Basal medium (Promo Cell GmbH, Heidelberg, Germany) according to the manufacturer’s recommendations. All experiments were performed with three technical replicates per condition, and each experiment was independently repeated in iPSC-EC from six male and six female donors (biological replicates). Technical replicates refer to repeated measurements within the same donor and condition, whereas biological replicates represent independent donor-level samples used for statistical analysis.

### 2.2 Treatment of iPSC-EC

A total of 5 × 10^5^ iPSC-EC were seeded per well into 6-well plates and cultured to confluence. To induce a pro-atherogenic phenotype, cells were first treated with 10 ng/ml TNF-α (Invitrogen, Darmstadt, Germany) for 24 h, a concentration widely used to elicit robust endothelial activation *in vitro* (11). This was followed by an additional 24 h treatment with either 1 µg/ml resveratrol (Sigma-Aldrich, Taufkirchen, Germany) or 1 µg/ml metformin (Stemcell Technologies, Köln, Germany), doses within the range previously reported to modulate mitochondrial and inflammatory pathways in endothelial cells (27, 28). Untreated cells without vehicle served as basal control and vehicle control for water soluble metformin, and 0.1% DMSO (Roth, Karlsruhe, Germany) as vehicle control for resveratrol.

### 2.3 Mitochondrial superoxide measurements

Mitochondrial superoxide-associated signal was assessed in male and female iPSC-EC following TNF-α treatment for 24 h and subsequent resveratrol or metformin treatment for additional 24 h using MitoSOX Red (Catalog Nr. M36008; ThermoFisher Scientific, Darmstadt, Germany), according to the manufacturer’s instructions. Briefly, cells were incubated with 5 µmol/L MitoSOX Red for 30 min, washed twice with PBS containing 1 mmol/l CaCl_2_, and lysed in buffer (29, 30). Fluorescence was measured using a plate reader (Tecan, Männedorf, Switzerland) (excitation 485 ± 10 nm, emission 530 ± 10 nm). Total protein content of each sample was quantified by Pierce 660nm Protein assay (Catalog Nr. 22660, ThermoFisher Scientific, Darmstadt, Germany) and used for normalization to the total protein content of each sample.

### 2.4 Mitochondrial mass analysis

Mitochondrial DNA (mtDNA) and nuclear DNA (nDNA) content were quantified in male and female iPSC-EC by quantitative real-time PCR. Relative mitochondrial content was calculated as the ratio of mtDNA to nDNA. For detection of mtDNA, mt-RNR2-specific primers (forward 5’-CCACATCTGCCGAGACGTAA-3’, reverse 5’-TAGTCCTCGTCCCACATGGA-3’) were used, whereas nDNA was amplified using β-globin-specific primers (forward 5’-AAGTACCACTAAGCCCCCTTTC-3’, reverse 5’-GGGAACACAAAAGACCTCTTCTGG’) with SYBR Green. β-globin was chosen as nuclear reference gene because it is a single-copy nuclear locus that is widely used for normalization of mtDNA/nDNA ratios in cardiovascular tissues (31).

### 2.5 Flow cytometry

Adhesion molecule expression on male and female iPSC-EC treated with TNF-α and combination of TNF-α and resveratrol or metformin was assessed by flow cytometry as previously described (11). The following antibodies were used: human anti-CD106- PE-Cyanine7 (VCAM-1) (Clone: STA, Cat# 25-1069-42, ThermoFisher Scientific, Germany), human anti-CD54-Alexa-Fluor770 (ICAM-1) (Clone: HA58, Cat# 353126, BioLegend, Germany) and human anti-CD62E-PE (E-selectin) (Cat# 130-113-128, BioLegend, Germany). Flow cytometry acquisition was performed on an Attune NxT flow cytometer with blue, red and violet laser (Invitrogen, USA), and data were analyzed using Kaluza Analysis 2.1 software (Beckman Coulter, Germany).

### 2.6 Immunofluorescence

iPSC-EC, HUVEC and Eahy926 cells were cultivated in 8-chamber slides (Sigma-Aldrich, Germany). Cells were fixed with 4% Histofix (Roth, Germany) and permeabilized with 0.2% Triton X-100 (Sigma-Aldrich). Cells were stained against vWF (1:100, Dako, Germany) (**Supplementary Figure 1**). The secondary antibody anti-mouse FITC (1:100) (Dianova, Germany) was applied according to the manufacturer’s protocol. Nuclei were stained using DAPI (1:50000) (Sigma, Germany) and cells were mounted with Fluoromount G (Southern Biotech). Negative controls were performed by omitting the primary antibody. Images were acquired using a BZ-9000E fluorescence microscope (Keyence, Germany). All evaluations were performed in a blinded manner.

### 2.7 Seahorse assay

Male and female iPSC-EC were seeded at a density of 3 × 10^4^ cells per well in XF96 cell culture microplates (Agilent XFe96, Agilent Santa Clara, CA, USA). One well in each corner of the plate was filled with a supplemented medium without cells to serve as background control. After attachment, cells were treated as described above. A utility plate containing 200 μl/well of Seahorse XF calibrant and the corresponding sensor cartridge were hydrated overnight at 37°C in a non-CO_2_ incubator according to the manufacturer’s instructions. On the following day, the culture medium in the iPSC-EC plate was replaced with Seahorse XF assay medium (supplemented with 10 mmol/l glucose and 2 mmol/l glutamine), and cells were equilibrated for 1 h at 37°C in a non-CO_2_ incubator (29, 30).

For the Mito Stress Test (Agilent Santa Clara, CA, USA), injector ports were loaded with 22-25 μl of the following compounds at their final working concentrations: oligomycin (1 μmol/l; ATP synthase inhibitor to determine ATP-linked respiration), FCCP (0.75 μmol/l; mitochondrial uncoupler to assess maximal respiratory capacity), and a mixture of antimycin A (0.5 μmol/l) and rotenone (0.5 μmol/l; Complex III and I inhibitors to determine non-mitochondrial oxygen consumption). The assay was performed on a Seahorse XFe96 Analyzer (Agilent Santa Clara, CA, USA) following instrument calibration with the hydrated sensor cartridge. Oxygen consumption rate (OCR) and extracellular acidification rate (ECAR) were recorded over approximately 80 min, and all respiration parameters were normalized to sample protein content. After completion of the assay, cells were lysed in each well and total protein content was determined by Pierce 660nm Protein assay (Catalog Nr. 22660 ThermoFisher Scientific, Darmstadt, Germany). OCR and ECAR values were normalized to protein content per well. Three technical replicates and 6 biological replicates were performed.

### 2.8 Transwell permeability assay

Endothelial permeability of male and female iPSC-EC was assessed using the Endothelial Transwell Permeability Assay Kit (CB6929, Cell Biologics, Chicago, IL, USA) according to the manufacturer’s instructions. Briefly, 2.0 × 10 (5) iPSC-EC were seeded per insert onto collagen-coated polycarbonate Transwell filters (0.4 µm pore size) and cultured until a confluent monolayer was established. After the indicated treatments, biotinylated tracer and streptavidin-HRP solution were added to the upper chamber, and tracer flux into the lower chamber was quantified by measuring absorbance at 450 nm using a microplate reader (Tecan, Männedorf, Switzerland) within 1 h. Relative endothelial permeability was calculated after normalization to untreated control wells, as previously described by Chen et al. (32).

### 2.9 Caspase Glo assay

To assess caspase activity in male and female activated iPSC-EC after treatment with resveratrol or metformin, the luminogenic caspase-1 substrate Z-WEHD-aminoluciferin (Promega, Walldorf, Germany) was added to the cells according to the manufacturer’s instructions. To exclude non-specific proteasome-mediated cleavage of the substrate, the proteasome inhibitor MG-132 was included. After incubation of the plate for 1 h at room temperature, luminescence was measured using a luminometer (Tecan, Männedorf, Switzerland).

### 2.10 Determination of lactate dehydrogenase activity

Lactate dehydrogenase (LDH) activity was determined in the supernatant of treated and untreated female and male iPSC-EC using the Cytotoxicity Detection KitPLUS (Roche Applied Science, Penzberg, Germany), according to the manufacturer’s instructions. Absorbance was measured with a microplate reader (Tecan, Männedorf, Switzerland), and LDH activity was calculated as an indicator of cell membrane integrity and cytotoxicity.

### 2.11 ELISA

Supernatants from untreated and TNF-α–activated male and female iPSC-EC treated with resveratrol or metformin were collected, and ELISA assays for IL-6 (1:200, BioLegend, San Diego, CA, USA), TNF-α (1:200, BioLegend, San Diego, CA, USA), IL-10 (1:200, BioLegend, San Diego, CA, USA), and MCP-1 (1:200, BioLegend, San Diego, CA, USA) were performed according to the manufacturer’s instructions.

### 2.12 Statistical analysis

All data are presented as mean ± standard error of the mean (SEM), where means refer to biological replicates (individual donors; n=6). For each donor and condition, 3 technical replicates were averaged to obtain one biological replicate, and these donor-level means are shown as individual data points in the figures. Exploratory two-way ANOVA models with sex and treatment as fixed factors were calculated for selected endpoints to examine overall sex- and treatment effects and potential interactions; however, most effects did not remain statistically significant after correction for multiple comparisons, and these ANOVA results are therefore interpreted in a descriptive, hypothesis-generating manner only. All statistical test performed were two-sided, and p-values < 0.05 were considered statistically significant; effects with higher p-values < 0.1 are reported as non-significant trends. In addition, for some endpoints pairwise comparisons were explored using non-parametric tests; their results are reported in the figure annotations and described as exploratory trends.

## 3 Results

### 3.1 Sex-associated patterns of resveratrol and metformin on MitoSOX signal and endothelial permeability in TNF-α–activated iPSC-EC

TNF-α treatment activates endothelial cells, increases ROS production and endothelial permeability, and thereby contributes to endothelial dysfunction (33–35). To investigate the effects of resveratrol and metformin on endothelial function, ROS formation, mitochondrial mass and endothelial permeability were assessed in TNF-α–activated male and female iPSC-EC.

**Figure 1:**
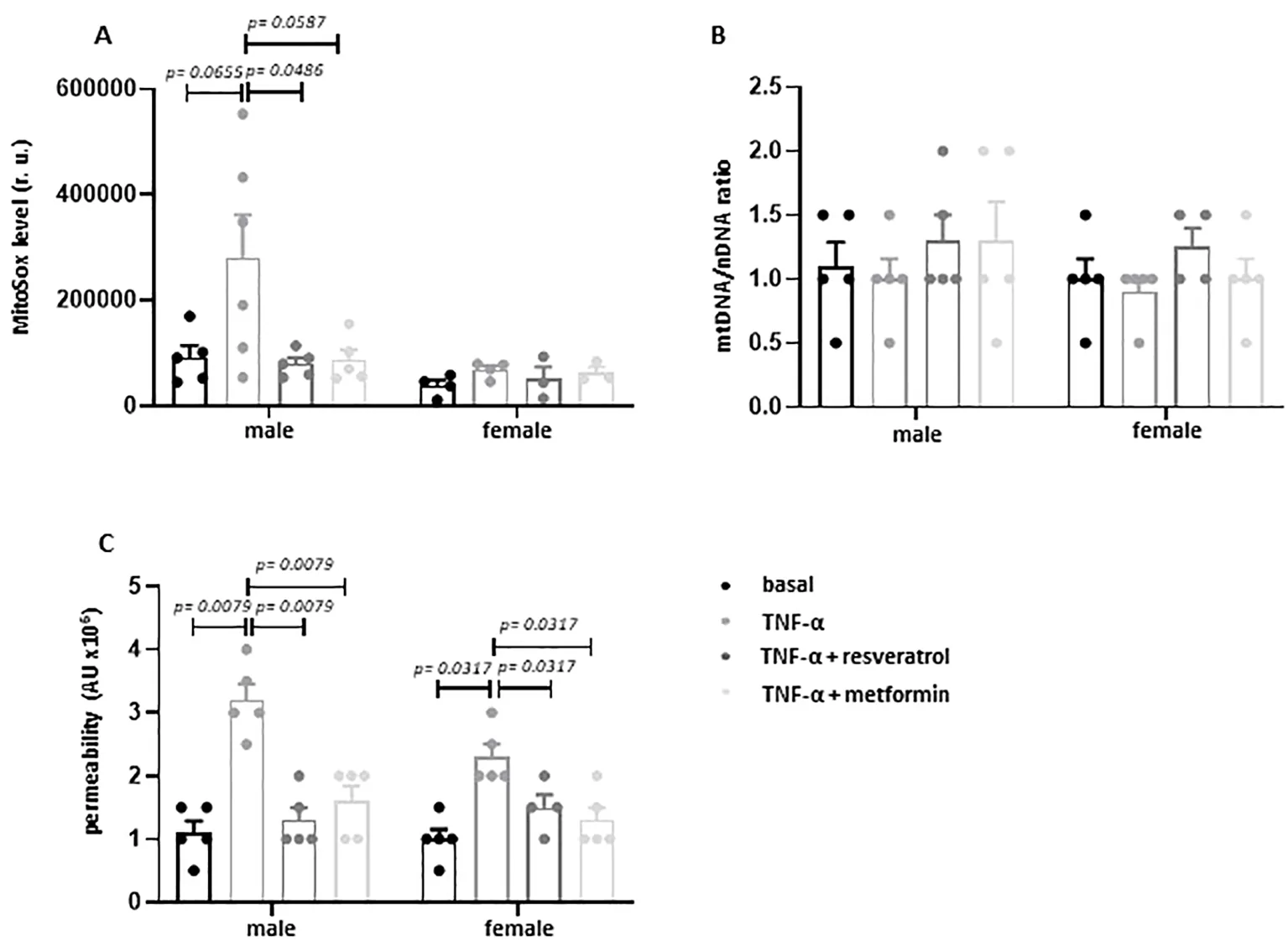
Sex-specific effects of TNF-α, resveratrol and metformin on ROS formation, mitochondrial mass and endothelial permeability in iPSC-EC. **(A)** Mitochondrial superoxide-associated signal in male and female iPSC-EC under basal conditions and after TNF-α stimulation (10 ng/ml, 24 h) followed by treatment with resveratrol (1 µg/ml) or metformin (1 µg/ml) for an additional 24 h. ROS were quantified using MitoSOX and normalized to protein content. **(B)** Mitochondrial mass expressed as the mtDNA/nDNA ratio in male and female iPSC-EC under the indicated conditions. mtDNA was quantified using mt-RNR2-specific primers and normalized to β-globin as nuclear reference. **(C)** Endothelial permeability of male and female iPSC-EC monolayers assessed in Transwell assays after TNF-α (10 ng/ml, 24 h) with or without resveratrol (1 µg/ml, 24 h) or metformin (1 µg/ml, 24 h). Data are presented as bar charts with individual data points and means ± SEM (n = 4–6 per group). All data were normalized to the mean of the male basal control. Statistical comparisons were performed using non-parametric tests, and effects with p ≥ 0.05 are reported as non-significant trends.

TNF-α stimulation significantly increased mitochondrial superoxide-associated signal in male iPSC-EC, whereas no effect was observed in female cells (p < 0.05 and p > 0.05, respectively; **Figure 1A**). In TNF-α-activated male iPSC-EC, both resveratrol and metformin significantly reduced mitochondrial superoxide-associated signal (p < 0.05; **Figure 1A**). Neither TNF-α nor resveratrol or metformin altered mitochondrial mass in iPSC-EC (**Figure 1B**). In contrast, TNF-α treatment significantly increased endothelial permeability in both male and female iPSC-EC (**Figure 1C**). Resveratrol and metformin significantly attenuated this effect, with a more pronounced reduction in male cells (p < 0.01 and p < 0.05, respectively; **Figure 1C**).

### 3.2 Sex-associated patterns of resveratrol and metformin on mitochondrial respiration in TNF-α–activated iPSC-EC

**Figure 2:**
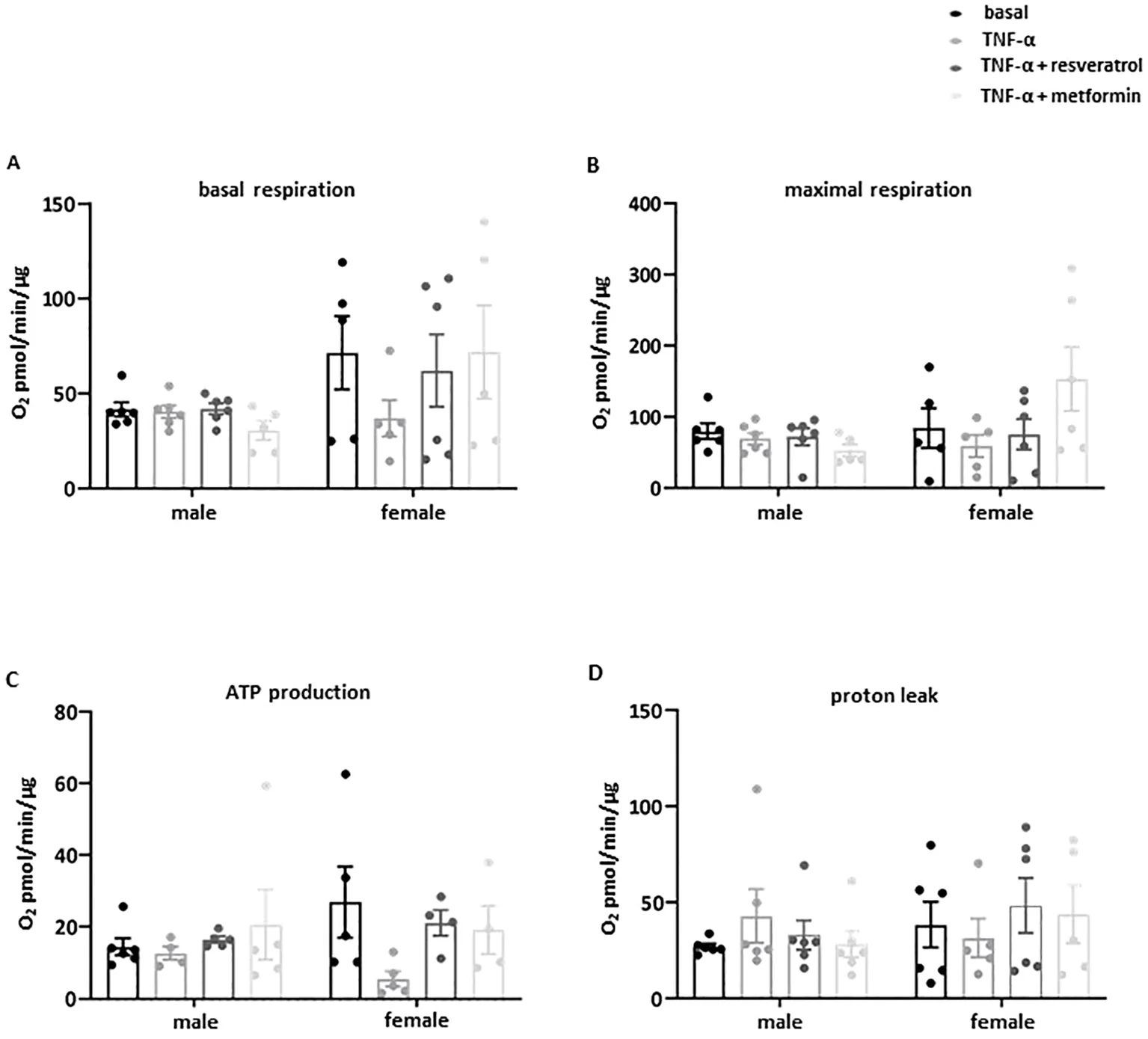
Sex-specific effects of resveratrol and metformin on mitochondrial respiration in TNF-α–activated iPSC-EC. Seahorse XF analyses of mitochondrial respiration parameters in male and female iPSC-EC under basal conditions and after TNF-α with or without resveratrol or metformin. **(A)** Basal respiration (O_2_ consumption rate, pmol/min/µg protein). **(B)** Maximal respiration after FCCP injection. **(C)** ATP-linked respiration calculated from oligomycin-sensitive OCR. **(D)** Proton leak OCR after oligomycin injection. Cells were stimulated with TNF-α (10 ng/ml, 24 h) followed by resveratrol (1 µg/ml, 24 h) or metformin (1 µg/ml, 24 h) before the Mito Stress Test (oligomycin, FCCP, antimycin A/rotenone) was performed. Data are shown as bar charts with individual data points and means ± SEM (n = 4–6 per group) and were normalized to the mean of the male basal control. Statistical comparisons were performed using non-parametric tests, and effects with p ≥ 0.05 are reported as non-significant trends.

To further investigate the effects of resveratrol and metformin on mitochondrial function, mitochondrial oxidative phosphorylation was analyzed. Under basal conditions, basal respiration showed a non-significant tendency to be lower in male iPSC-EC compared with female iPSC-EC (p > 0.05; **Figure 2A**). In female iPSC-EC, TNF-α exposure was associated with a non-significant decrease in basal respiration (p > 0.05), and subsequent treatment with resveratrol and metformin treatment showed a trend towards higher basal OCR compared with TNF-α alone, without reaching statistical significance (p > 0.05). No statistically significant changes in basal respiration were detected in male cells across treatments (**Figure 2A** and **Supplementary Figure 2A**). Consistent with this, TNF-α tended to reduce maximal respiration in female iPSC-EC, and metformin showed a non-significant tendency to increase maximal respiratory capacity (p > 0.05; **Figure 2B**; **Supplementary Figure 2A**). ATP-linked respiration and proton leak displayed non-significant decreases after TNF-α in female iPSC-EC, with resveratrol and metformin showing trends towards higher values compared with TNF-α alone, again without reaching statistical significance (p > 0.05). In male iPSC-EC, mitochondrial respiration parameters remained largely unchanged across conditions (p > 0.05; **Figures 2C, D**). ECAR showed a non-significant trend towards reduction after TNF-α treatment and partial recovery with metformin in both sexes (p> 0.05; **Supplementary Figure 2B**).

### 3.3 Sex-associated anti-inflammatory effects of resveratrol and metformin in TNF-α–activated iPSC-EC

**Figure 3:**
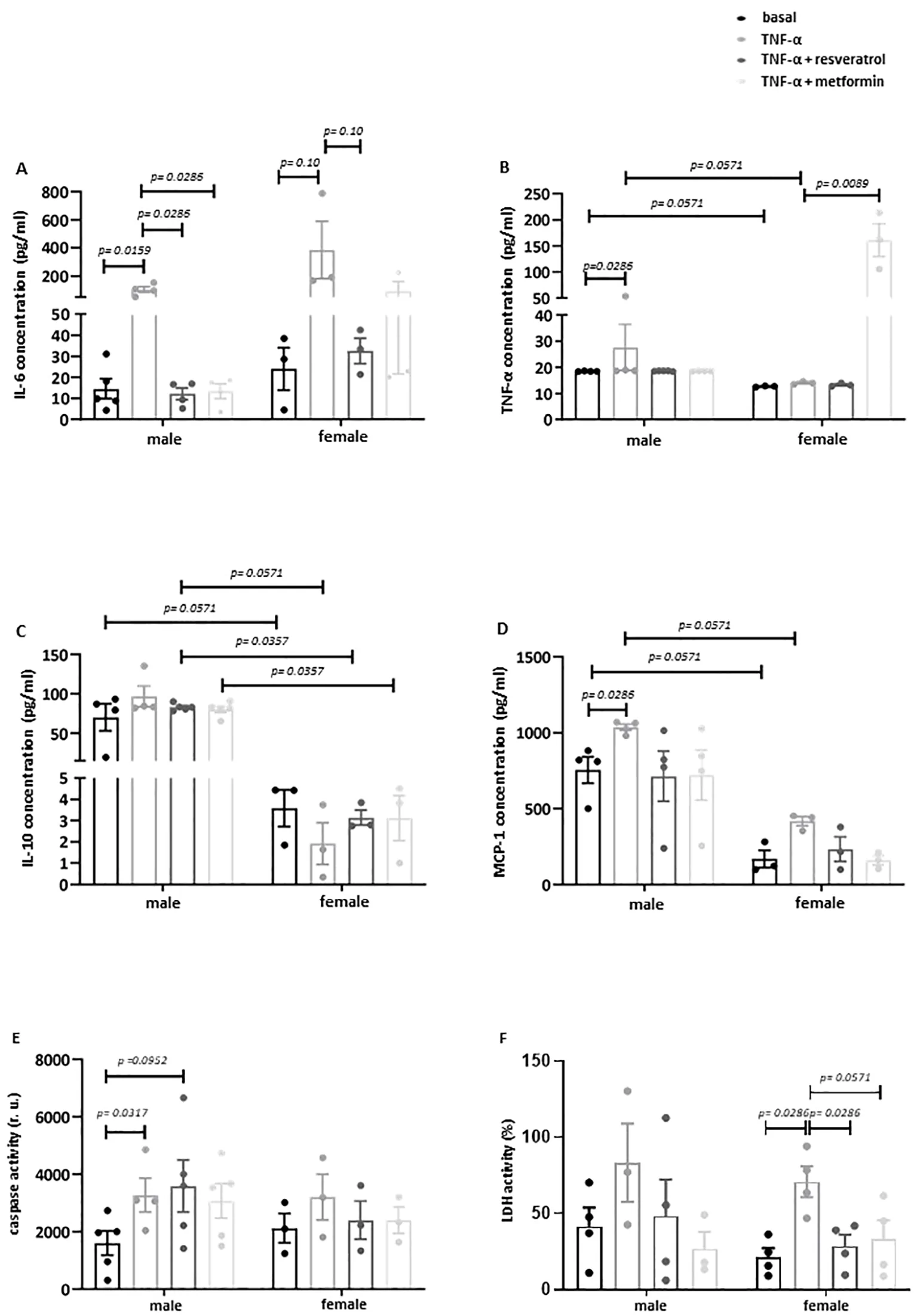
Sex-specific effects of resveratrol and metformin on inflammatory activation and cytotoxicity in TNF-α–treated iPSC-EC. ELISA quantification of IL-6 **(A)**, TNF-α **(B)**, IL-10 **(C)** and MCP-1 **(D)** concentrations (pg/ml) in supernatants from male and female iPSC-ECs after compounds treatment. **(E)** Caspase activity in male and female iPSC-ECs under basal conditions and after TNF-α with or without resveratrol or metformin. **(F)** LDH release in male and female iPSC-EC. Cells were pre-activated with TNF-α (10 ng/ml, 24 h) and subsequently treated with resveratrol (1 µg/ml, 24 h) or metformin (1 µg/ml, 24 h). Data are presented as bar charts with individual data points and means ± SEM (n = 4–6 per group). All values were normalized to the mean of the male basal control. r.u.: relative units. Statistical comparisons were performed using non-parametric tests, and effects with p ≥ 0.05 are reported as non-significant trends.

Since mitochondrial function is closely related to inflammation (36, 37), we next investigated the effects of resveratrol and metformin on inflammation in male and female activated iPSC-EC. TNF-α treatment increased the levels of IL-6 and MCP-1 in male and female iPSC-EC, with a more prominent effect in male cells (**Figures 3A, D**). Resveratrol and metformin treatment reduced the expression of both pro-inflammatory factors in both male and female activated cells (**Figures 3A, D**). The IL-6 levels were only increased in male iPSC-EC after TNF-α treatment and resveratrol and metformin attenuated this effect (**Figure 3A**). TNF-α levels were significantly increased only in male activated iPSC-EC, whereas no change was observed in female cells (**Figure 3B**). In male iPSC-EC, resveratrol and metformin reduced TNF-α concentrations back to basal levels (**Figure 3B**). Interestingly, co-treatment with metformin significantly increased TNF-α levels in female iPSC-EC (**Figure 3B**). In contrast, IL-10 levels were not affected in male cells after either of the treatments, while in female endothelial cells TNF-α tended to decrease IL-10 expression and resveratrol and metformin showed non-significant trends towards higher IL-10 expression compared to basal levels, but none of these changes reached statistical significance (p> 0.05; **Figure 3C**). In line with the expression of pro-inflammatory markers, TNF-α also increased caspase activity in male iPSC-EC, however neither resveratrol nor metformin abolished TNF-α effects in these cells (p> 0.05; **Figure 3E**), while caspase activity was not affected in female cells (p> 0.05; **Figure 3E**). TNF-α treatment increased LDH release in male and female endothelial cells, while resveratrol or metformin treatment showed modest non-significant tendencies towards reduced the LDH release in both male and female (**Figure 3F**), however the effects were more prominent in female iPSC-EC (**Figure 3F**).

**Figure 4:**
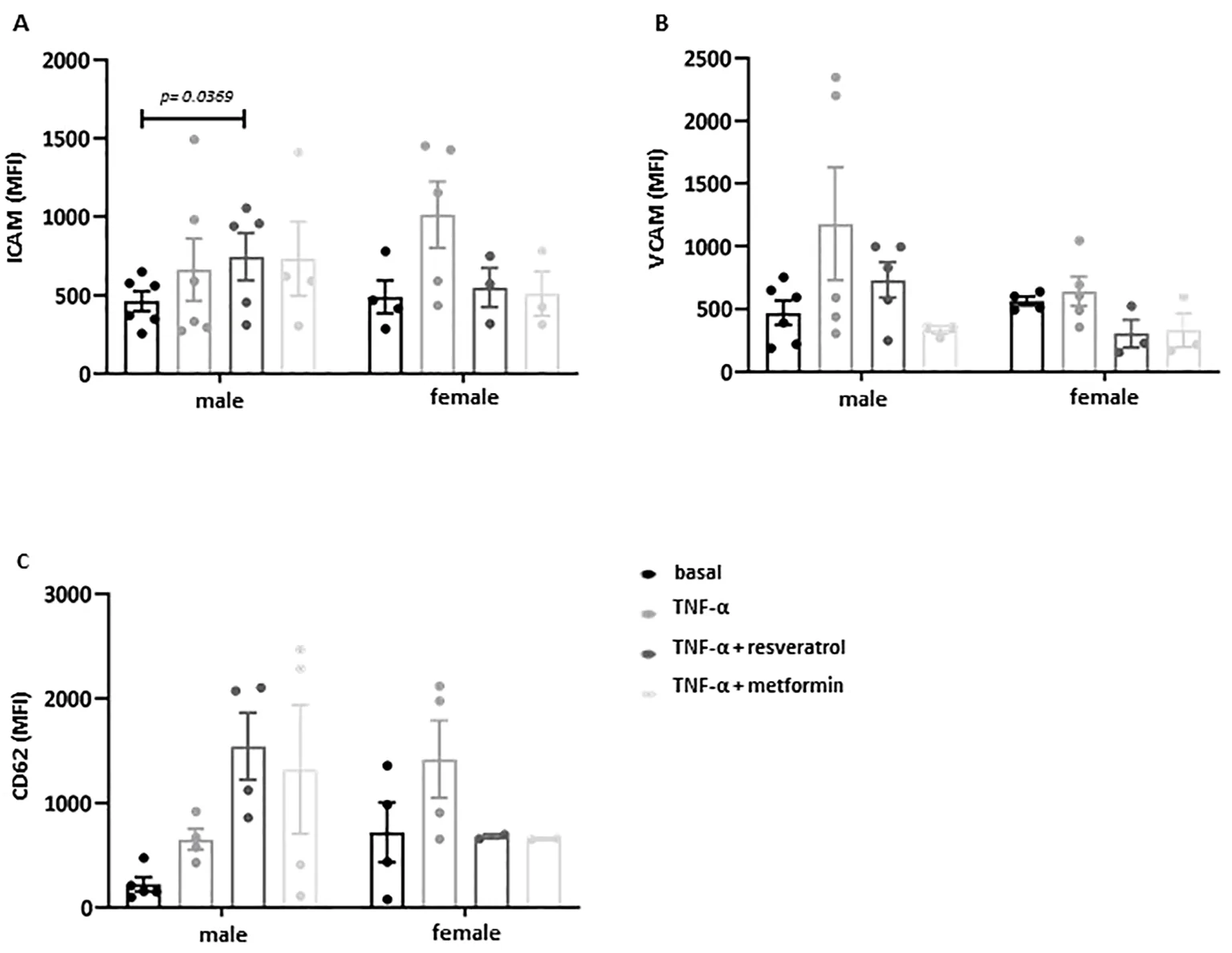
Effects of resveratrol and metformin on adhesion molecule expression in TNF-α–activated male and female iPSC-EC. Flow cytometry analyses of **(A)** ICAM-1, **(B)** VCAM-1 and **(C)** E-selectin (CD62) mean fluorescence intensity (MFI) in male and female iPSC-EC under basal conditions and after TNF-α with or without resveratrol or metformin. Cells were stimulated with TNF-α (10 ng/ml, 24 h) and then treated with resveratrol (1 µg/ml) or metformin (1 µg/ml) for 24 h. Data are shown as bar charts with individual data dots and means ± SEM (n = 4–6 per group) and were normalized to the mean of the male basal control. Statistical comparisons were performed using non-parametric tests, and effects with p ≥ 0.05 are reported as non-significant trends.

In addition, TNF-α induced modest, non-significant sex-associated patterns in adhesion molecule expression in iPSC-EC. In female endothelial cells, TNF-α tended to increase ICAM-1 and CD62 expression, whereas in male cells TNF-α predominantly tended to increase VCAM-1 (**Figures 4A–C**, p > 0.05). Resveratrol and metformin treatment showed a non-significant tendency to attenuate the TNF-α-induced upregulation of adhesion molecules in both sexes (**Figures 4A–C**, p > 0.05).

## 4 Discussion

In this study, we show that resveratrol and metformin exert sex-associated differences in selected responses in human iPSC-derived endothelial cells to TNF-α–driven inflammatory stress. Consistent with our previous work and other reports on sex-biased endothelial biology, TNF-α induced a more pronounced pro-oxidative and pro-inflammatory phenotype in male iPSC-EC, whereas female cells exhibited a pattern characterized by trends towards impaired mitochondrial respiration and lower anti-inflammatory IL-10. Resveratrol and metformin attenuated TNF-α–induced mitochondrial superoxide production and TNF-associated permeability increase in both sexes. For mitochondrial respiration parameters, these compounds mainly showed non-significant tendencies towards higher basal and maximal OCR, ATP-linked respiration and proton leak in female iPSC-EC, while respiration in male cells remained largely unchanged, supporting the concept that cellular sex critically shapes the balance between oxidative damage and mitochondrial compensation under inflammatory stress (38, 39). At the mitochondrial level, Seahorse analyses suggested that TNF-α tended to reduce basal and maximal respiration, ATP production and proton leak in female iPSC-EC, while male cells maintained overall similar mitochondrial respiration despite increased ROS. Treatment with resveratrol or metformin was associated with trends towards higher basal and maximal respiration and towards partial recovery of ATP production and proton leak in TNF-α–treated female cells, although these changes did not reach statistical significance in either sex. These findings are in line with previous work showing that mitochondrial function and autophagy exhibit sex differences (38, 40, 41), with female cells often showing greater reliance on mitochondrial quality control pathways and a differential response to metabolic stressors (40). In our model, resveratrol and metformin appear to preferentially support mitochondrial resilience in female iPSC-EC, whereas their dominant effect in male cells is to limit oxidative damage and endothelial barrier disruption.

In male iPSC-EC, TNF-α robustly increased ROS formation, endothelial permeability, IL-6 and MCP-1 secretion, caspase activity and LDH release, whereas these effects were less pronounced or absent in female cells. Resveratrol and metformin significantly reduced ROS levels, cytokine release, caspase activity and LDH in male cells, indicating a strong anti-oxidative, anti-inflammatory and cytoprotective profile in this sex (20, 23). In female iPSC-EC, TNF-α primarily decreased IL-10 and impaired mitochondrial respiration, and both resveratrol and metformin restored IL-10 levels and respiratory parameters towards baseline. This pattern suggests that in male endothelial cells, resveratrol and metformin mainly counteract an exaggerated pro-inflammatory and pro-apoptotic response, while in female cells they help maintain mitochondrial function and an anti-inflammatory milieu. The observed sex-specific effects integrate well with current concepts of sex differences in cardiovascular aging and endothelial biology (42). Male vascular cells tend to develop a more pronounced pro-inflammatory and pro-oxidative phenotype under stress (43, 44), whereas female cells exhibit better preserved mitochondrial function and antioxidant capacity at younger ages but (45) may be more vulnerable to mitochondrial dysfunction and autophagy defects with aging and loss of estrogen (14, 46, 47), although age-related changes and donor heterogeneity were not formally analyzed in our cohort. Our data extend these concepts to an iPSC-EC model and highlight that resveratrol and metformin can differentially modulate these sex-biased trajectories at the cellular level: limiting ROS and cell injury in male cells, and stabilizing mitochondrial respiration and IL-10 production in female cells.

Furthermore, we observed sex-associated patterns of endothelial adhesion molecules in response to TNF-α and cardiometabolic modulation, in line with prior reports. Hunter et al. demonstrated that human brain microvascular endothelial cells from male and female donors exhibit distinct TNF-α–induced patterns of ICAM-1, VCAM-1 and E-selectin on endothelial microvesicles (48), supporting the concept that cellular sex shapes the endothelial adhesion profile under inflammatory stress. Recent transcriptomic and mechanistic studies further highlight sex-dependent endothelial TNF-α/NF-κB signaling and adhesion molecule gene regulation (12, 48), consistent with our finding that TNF-α preferentially increases ICAM-1 and E-selectin in female iPSC-EC, whereas VCAM-1 upregulation is more prominent in male cells. Our data that resveratrol and metformin attenuate TNF-α–induced ICAM-1, VCAM-1 and E-selectin expression in both sexes are also well supported by the literature. Resveratrol has been shown to prevent TNF-α–induced upregulation of VCAM-1, ICAM-1 and E-selectin and to reduce monocyte adhesion to endothelial(-progenitor) cells via inhibition of NF-κB signaling (49, 50). Similarly, metformin decreases hyperglycemia-induced expression of ICAM-1, VCAM-1 and E-selectin in human endothelial cells, at least in part through AMPK-dependent suppression of pro-inflammatory pathways (23, 51).

Together with these studies, our results suggest that resveratrol and metformin converge on shared anti-inflammatory mechanisms that limit leukocyte–endothelium interactions, while the precise adhesion molecule pattern remains modified by cellular sex.

Clinically, these findings support the notion that polyphenols and metabolic modulators may exert their benefits in a sex-dependent manner (52, 53). Resveratrol is widely promoted as a general antioxidant and longevity compound (54, 55), yet our data suggest that its protective profile may differ in male vs. female endothelium, particularly under inflammatory conditions. Similarly, metformin – beyond its glucose-lowering effects – is increasingly discussed as a cardiovascular and geroprotective agent (24, 56); our results indicate that its endothelial benefits might involve sex-specific modulation of mitochondrial respiration and inflammatory signaling. While our study is preclinical, using iPSC-derived cells, it underscores the importance of considering cellular sex as a biological variable when evaluating vascular protective therapies and may help generate hypotheses for sex-stratified clinical studies.

## 5 Limitations

Several limitations should be acknowledged. Our experiments were performed in iPSC-EC from a limited number of donors; larger cohorts will be needed to capture inter-individual variability. In addition, some endpoints showed substantial variability, which likely reflects inter-donor heterogeneity inherent to human iPSC-derived models as well as technical variability of complex assays such as Seahorse measurements. While this variability limits the power to detect small effects, it also captures biologically relevant donor-to-donor differences. Consistent with this, exploratory two-way analyses including sex and treatment as factors did not reveal robust statistically significant interaction effects, underscoring that our study should be regarded as exploratory and hypothesis-generating rather than confirmatory. Larger cohorts of iPSC-EC from male and female donors will be required to validate these sex-associated patterns and to refine the statistical modelling. Furthermore, we focused on acute TNF-α exposure and short-term resveratrol/metformin treatment; chronic exposure and dose–response relationships were not addressed. Finally, we did not directly interrogate upstream signaling pathways (e.g. Sirt1, AMPK, autophagy markers) that might mechanistically link resveratrol and metformin to the observed functional effects. Future work should combine this iPSC-EC platform with detailed analyses of sirtuin/AMPK signaling, mitophagy, autophagy flux and senescence markers, ideally in parallel with primary endothelial cells from older patients.

## 6 Conclusion

Our study demonstrates that resveratrol and metformin exert distinct, sex-specific protective effects in a TNF-α–driven iPSC-EC inflammatory dysfunction model. Male iPSC-EC predominantly benefit from reduced oxidative stress, inflammation and markers of cell injury, whereas female cells show tendencies towards improved mitochondrial respiration and higher anti-inflammatory IL-10 in response to both compounds. These results emphasize cellular sex as a critical determinant of endothelial responses to inflammatory stress and to vascular protective therapies, and support the development of sex-aware strategies for targeting mitochondrial dysfunction and endothelial cell activation in early atherosclerosis.

## Author’s note

Human iPSC lines were kindly provided by Dr. Elise Kessler and Prof. Dr. Joost Sluijter, University Medical Center Utrecht, the Netherlands.

## Data Availability

The original contributions presented in the study are included in the article/**Supplementary Material**. Further inquiries can be directed to the corresponding author.

## Supplementary material

Supplementary Figure 1 iPSC-EC are positive for vWF. Representative images of HUVEC, EA.hy926 and Ipsc-EC cells stained against vWF (green). Nuclei were stained with DAPI (blue). Magnification: 40x, scale bar: 50 µm.

Supplementary Figure 2 Mitochondrial respiration and glycolysis in male and female iPSC-EC cells under different treatment conditions. (A) Oxygen consumption rate (OCR, pmol/min) as a measure of mitochondrial respiration was recorded over time in male (left) and female (right) cells using a Seahorse assay. After baseline measurements, sequential injections of mitochondrial modulators were applied as indicated, and OCR traces are shown for the different treatment groups. (B) Extracellular acidification rate (ECAR, mpH/min) as a surrogate for glycolytic activity was measured in parallel in male (left) and female (right) cells over time. Cells were stimulated with TNF-α (10 ng/ml, 24 h) and then treated with resveratrol (1 µg/ml) or metformin (1 µg/ml) for 24 h (n = 6 per group). Statistical comparisons were performed using non-parametric tests, and effects with p ≥ 0.05 are reported as non-significant trends.

## References

[1] Roth GA Mensah GA Johnson CO Addolorato G Ammirati E Baddour LM . Global burden of cardiovascular diseases and risk factors, 1990-2019: Update from the GBD 2019 study. J Am Coll Cardiol. (2020) 76:2982–3021. doi: 10.1016/j.jacc.2020.11.010 33309175 PMC7755038

[2] Wang Y Wang X Wang C Zhou J . Global, regional, and national burden of cardiovascular disease, 1990-2021: Results from the 2021 global burden of disease study. Cureus. (2024) 16:e74333. doi: 10.7759/cureus.74333 39720386 PMC11668263

[3] Hakamaa E Goebeler S Martiskainen M Louhelainen AM Ahinko K Lehtimaki T . Sex differences in coronary atherosclerosis during the pre- and postmenopausal period: The Tampere sudden death study. Atherosclerosis. (2024) 390:117459. doi: 10.1016/j.atherosclerosis.2024.117459 38364347

[4] Laban D Kattan A Ait-Abdellah L Krishna H Le Master E Levitan I . Sex differences in features of atherosclerotic plaques as revealed by various imaging techniques: Historical review. Front Physiol. (2025) 16:1579885. doi: 10.3389/fphys.2025.1579885 40491450 PMC12146876

[5] Chrysohoou C Iliakis P Pitsillidi A Manta E Barkas F Liberopoulos E . Twenty-year cardiovascular disease incidence in menopausal women: Insights from the ATTICA study. Climacteric. (2026) 29:231–8. doi: 10.1080/13697137.2025.2567691 41122785

[6] Davignon J Ganz P . Role of endothelial dysfunction in atherosclerosis. Circulation. (2004) 109:III27–32. doi: 10.1161/01.cir.0000131515.03336.f8 15198963

[7] DiCorleto PE Chisolm GM 3rd . Participation of the endothelium in the development of the atherosclerotic plaque. Prog Lipid Res. (1986) 25:365–74. doi: 10.1016/0163-7827(86)90074-3 3321089

[8] Marui N Offermann MK Swerlick R Kunsch C Rosen CA Ahmad M . Vascular cell adhesion molecule-1 (VCAM-1) gene transcription and expression are regulated through an antioxidant-sensitive mechanism in human vascular endothelial cells. J Clin Invest. (1993) 92:1866–74. doi: 10.1172/jci116778 7691889 PMC288351

[9] Gimbrone MA Jr. Garcia-Cardena G . Endothelial cell dysfunction and the pathobiology of atherosclerosis. Circ Res. (2016) 118:620–36. doi: 10.1161/circresaha.115.306301 26892962 PMC4762052

[10] Cattaneo MG Banfi C Brioschi M Lattuada D Vicentini LM . Sex-dependent differences in the secretome of human endothelial cells. Biol Sex Differ. (2021) 12:7. doi: 10.1186/s13293-020-00350-3 33413676 PMC7791663

[11] Lorenz M Palant R Oscherowa E Karmid-Haj Hamoud W Kirwan JA Trajkovski S . Pro-inflammatory activation promotes atherogenic endothelial phenotype in male and female human umbilical endothelial vein cells (HUVECs). Int J Mol Sci. (2026) 27. doi: 10.3390/ijms27073079 41977266 PMC13073718

[12] Shin J Hong J Edwards-Glenn J Krukovets I Tkachenko S Adelus ML . Unraveling the role of sex in endothelial cell dysfunction: Evidence from lineage tracing mice and cultured cells. Arterioscler Thromb Vasc Biol. (2024) 44:238–53. doi: 10.1161/atvbaha.123.319833 38031841 PMC10842863

[13] Jia H Moore M Wadhwa M Burns C . Human iPSC-derived endothelial cells exhibit reduced immunogenicity in comparison with human primary endothelial cells. Stem Cells Int. (2024) 2024:6153235. doi: 10.1155/sci/6153235 39687754 PMC11649354

[14] Barcena de Arellano ML Pozdniakova S Kuhl AA Baczko I Ladilov Y Regitz-Zagrosek V . Sex differences in the aging human heart: Decreased sirtuins, pro-inflammatory shift and reduced anti-oxidative defense. Aging (Albany NY). (2019) 11:1918–33. doi: 10.18632/aging.101881 30964749 PMC6503880

[15] Dela Justina V Miguez JSG Priviero F Sullivan JC Giachini FR Webb RC . Sex differences in molecular mechanisms of cardiovascular aging. Front Aging. (2021) 2:725884. doi: 10.3389/fragi.2021.725884 35822017 PMC9261391

[16] Ungvari A Gulej R Patai R Papp Z Toth A Szabo AA . Sex-specific mechanisms in vascular aging: Exploring cellular and molecular pathways in the pathogenesis of age-related cardiovascular and cerebrovascular diseases. Geroscience. (2025) 47:301–37. doi: 10.1007/s11357-024-01489-2 39754010 PMC11872871

[17] Sulaiman M Matta MJ Sunderesan NR Gupta MP Periasamy M Gupta M . Resveratrol, an activator of SIRT1, upregulates sarcoplasmic calcium ATPase and improves cardiac function in diabetic cardiomyopathy. Am J Physiol Heart Circ Physiol. (2010) 298:H833–843. doi: 10.1152/ajpheart.00418.2009 20008278 PMC2838561

[18] Li YG Zhu W Tao JP Xin P Liu MY Li JB . Resveratrol protects cardiomyocytes from oxidative stress through SIRT1 and mitochondrial biogenesis signaling pathways. Biochem Biophys Res Commun. (2013) 438:270–6. doi: 10.1016/j.bbrc.2013.07.042 23891692

[19] Kao CL Chen LK Chang YL Yung MC Hsu CC Chen YC . Resveratrol protects human endothelium from H(2)O(2)-induced oxidative stress and senescence via SirT1 activation. J Atheroscler Thromb. (2010) 17:970–9. doi: 10.5551/jat.4333 20644332

[20] Csiszar A Labinskyy N Podlutsky A Kaminski PM Wolin MS Zhang C . Vasoprotective effects of resveratrol and SIRT1: Attenuation of cigarette smoke-induced oxidative stress and proinflammatory phenotypic alterations. Am J Physiol Heart Circ Physiol. (2008) 294:H2721–2735. doi: 10.1152/ajpheart.00235.2008 18424637 PMC2551743

[21] Csiszar A Labinskyy N Pinto JT Ballabh P Zhang H Losonczy G . Resveratrol induces mitochondrial biogenesis in endothelial cells. Am J Physiol Heart Circ Physiol. (2009) 297:H13–20. doi: 10.1096/fasebj.24.1_supplement.775.8 PMC2711732 19429820

[22] Wu Y Li X Zhu JX Xie W Le W Fan Z . Resveratrol-activated AMPK/SIRT1/autophagy in cellular models of Parkinson's disease. Neurosignals. (2011) 19:163–74. doi: 10.1159/000328516 21778691 PMC3699815

[23] Hattori Y Suzuki K Hattori S Kasai K . Metformin inhibits cytokine-induced nuclear factor kappaB activation via AMP-activated protein kinase activation in vascular endothelial cells. Hypertension. (2006) 47:1183–8. doi: 10.1161/01.hyp.0000221429.94591.72 16636195

[24] Wang Q Zhang M Torres G Wu S Ouyang C Xie Z . Metformin suppresses diabetes-accelerated atherosclerosis via the inhibition of Drp1-mediated mitochondrial fission. Diabetes. (2017) 66:193–205. doi: 10.2337/db16-0915 27737949 PMC5204316

[25] Fontanelli L Castronovo A Ferri C Vozzi F Recchia FA Borghini A . iPSC-derived endothelial cells as experimental models for predictive and personalized strategies in cardiovascular and cerebrovascular disease. Int J Mol Sci. (2026) 27. doi: 10.20944/preprints202512.2490.v1 PMC12841387 41596430

[26] Patsch C Challet-Meylan L Thoma EC Urich E Heckel T O'Sullivan JF . Generation of vascular endothelial and smooth muscle cells from human pluripotent stem cells. Nat Cell Biol. (2015) 17:994–1003. doi: 10.1038/ncb3205 26214132 PMC4566857

[27] LaMoia TE Shulman GI . Cellular and molecular mechanisms of metformin action. Endocr Rev. (2021) 42:77–96. doi: 10.1210/endrev/bnaa023 32897388 PMC7846086

[28] Wallerath T Deckert G Ternes T Anderson H Li H Witte K . Resveratrol, a polyphenolic phytoalexin present in red wine, enhances expression and activity of endothelial nitric oxide synthase. Circulation. (2002) 106:1652–8. doi: 10.1161/01.cir.0000029925.18593.5c 12270858

[29] Samit LH Temp J Flores IA Vakhrusheva O Ladilov Y Barcena ML . Male monocyte-like cells are prone to a senescence-induced pro-inflammatory state. Aging Dis. (2025). doi: 10.14336/AD.2025.0871 41296928

[30] Barcena ML Christiansen-Mensch C Aslam M Haritonow N Ladilov Y Regitz-Zagrosek V . Upregulation of mitochondrial Sirt3 and alleviation of the inflammatory phenotype in macrophages by estrogen. Cells. (2024) 13. doi: 10.3390/cells13171420 39272992 PMC11393879

[31] Barcena ML Tonini G Haritonow N Breiter P Milting H Baczko I . Sex and age differences in AMPK phosphorylation, mitochondrial homeostasis, and inflammation in hearts from inflammatory cardiomyopathy patients. Aging Cell. (2023) 22:e13894. doi: 10.1111/acel.13894 37365150 PMC10410062

[32] Chen HR Yeh TM . *In vitro* assays for measuring endothelial permeability by transwells and electrical impedance systems. Bio Protoc. (2017) 7:e2273. doi: 10.21769/bioprotoc.2273 34541256 PMC8410333

[33] Gao X Zhang H Belmadani S Wu J Xu X Elford H . Role of TNF-alpha-induced reactive oxygen species in endothelial dysfunction during reperfusion injury. Am J Physiol Heart Circ Physiol. (2008) 295:H2242–2249. doi: 10.1152/ajpheart.00587.2008 18849334 PMC2614540

[34] Picchi A Gao X Belmadani S Potter BJ Focardi M Chilian WM . Tumor necrosis factor-alpha induces endothelial dysfunction in the prediabetic metabolic syndrome. Circ Res. (2006) 99:69–77. doi: 10.1161/01.res.0000229685.37402.80 16741160

[35] Royall JA Berkow RL Beckman JS Cunningham MK Matalon S Freeman BA . Tumor necrosis factor and interleukin 1 alpha increase vascular endothelial permeability. Am J Physiol. (1989) 257:L399–410. doi: 10.1152/ajplung.1989.257.6.l399 2610269

[36] Jin HS Suh HW Kim SJ Jo EK . Mitochondrial control of innate immunity and inflammation. Immune Netw. (2017) 17:77–88. doi: 10.4110/in.2017.17.2.77 28458619 PMC5407986

[37] Marchi S Guilbaud E Tait SWG Yamazaki T Galluzzi L . Mitochondrial control of inflammation. Nat Rev Immunol. (2023) 23:159–73. doi: 10.1038/s41577-022-00760-x 35879417 PMC9310369

[38] Demarest TG McCarthy MM . Sex differences in mitochondrial (dys)function: Implications for neuroprotection. J Bioenerg Biomembr. (2015) 47:173–88. doi: 10.1007/s10863-014-9583-7 25293493 PMC4988325

[39] Okoye DO Aboagye E Swenson S Ngugi S Shay K Ogola BO . Sex differences and oxidative stress in cardiovascular disease. Physiol (Bethesda). (2026). doi: 10.1152/physiol.00039.2025 41874226 PMC13092227

[40] Luk HY Jiwan NC Appell CR Levitt DE Vingren JL . Sex-specific mitochondrial dynamics and mitophagy response to muscle damage. Physiol Rep. (2022) 10:e15230. doi: 10.14814/phy2.15230 35611770 PMC9131605

[41] Triolo M Oliveira AN Kumari R Hood DA . The influence of age, sex, and exercise on autophagy, mitophagy, and lysosome biogenesis in skeletal muscle. Skelet Muscle. (2022) 12:13. doi: 10.1186/s13395-022-00296-7 35690879 PMC9188089

[42] Ji H Kwan AC Chen MT Ouyang D Ebinger JE Bell SP . Sex differences in myocardial and vascular aging. Circ Res. (2022) 130:566–77. doi: 10.1161/circresaha.121.319902 35175845 PMC8863105

[43] Huxley VH Kemp SS Schramm C Sieveking S Bingaman S Yu Y . Sex differences influencing micro- and macrovascular endothelial phenotype *in vitro*. J Physiol. (2018) 596:3929–49. doi: 10.1113/jp276048 29885204 PMC6117589

[44] Robert J . Sex differences in vascular endothelial cells. Atherosclerosis. (2023) 384:117278. doi: 10.1016/j.atherosclerosis.2023.117278 37770334

[45] Ventura-Clapier R Moulin M Piquereau J Lemaire C Mericskay M Veksler V . Mitochondria: A central target for sex differences in pathologies. Clin Sci (Lond). (2017) 131:803–22. doi: 10.1042/cs20160485 28424375

[46] Bajbouj K Shafarin J Taneera J Hamad M . Estrogen signaling induces mitochondrial dysfunction-associated autophagy and senescence in breast cancer cells. Biol (Basel). (2020) 9. doi: 10.3390/biology9040068 32244623 PMC7235898

[47] Sasaki Y Ikeda Y Uchikado Y Akasaki Y Sadoshima J Ohishi M . Estrogen plays a crucial role in Rab9-dependent mitochondrial autophagy, delaying arterial senescence. J Am Heart Assoc. (2021) 10:e019310. doi: 10.1161/jaha.120.019310 33719502 PMC8174372

[48] Hunter LW Jayachandran M Miller VM . Sex differences in the expression of cell adhesion molecules on microvesicles derived from cultured human brain microvascular endothelial cells treated with inflammatory and thrombotic stimuli. Biol Sex Differ. (2019) 10:26. doi: 10.1186/s13293-019-0241-y 31118073 PMC6532199

[49] Kim DS Kwon HM Choi JS Kang SW Ji GE Kang YH . Resveratrol blunts tumor necrosis factor-alpha-induced monocyte adhesion and transmigration. Nutr Res Pract. (2007) 1:285–90. doi: 10.4162/nrp.2007.1.4.285 20368952 PMC2849036

[50] Zhang Y Liu H Tang W Qiu Q Peng J . Resveratrol prevents TNF-alpha-induced VCAM-1 and ICAM-1 upregulation in endothelial progenitor cells via reduction of NF-kappaB activation. J Int Med Res. (2020) 48:300060520945131. doi: 10.1177/0300060520945131 32924701 PMC7493253

[51] Ding Y Zhou Y Ling P Feng X Luo S Zheng X . Metformin in cardiovascular diabetology: a focused review of its impact on endothelial function. Theranostics. (2021) 11:9376–96. doi: 10.7150/thno.64706 34646376 PMC8490502

[52] Louis XL Raj P Chan L Zieroth S Netticadan T Wigle JT . Are the cardioprotective effects of the phytoestrogen resveratrol sex-dependent? (1). Can J Physiol Pharmacol. (2019) 97:503–14. doi: 10.1139/cjpp-2018-0544 30576226

[53] Kwok MK Schooling CM . Unraveling potential sex-specific effects of cardiovascular medications on longevity using Mendelian randomization. J Am Heart Assoc. (2023) 12:e030943. doi: 10.1161/jaha.123.030943 38108247 PMC10863757

[54] Gal R Deres L Toth K Halmosi R Habon T . The effect of resveratrol on the cardiovascular system from molecular mechanisms to clinical results. Int J Mol Sci. (2021) 22. doi: 10.3390/ijms221810152 34576315 PMC8466271

[55] Zhou DD Luo M Huang SY Saimaiti A Shang A Gan RY . Effects and mechanisms of resveratrol on aging and age-related diseases. Oxid Med Cell Longev. (2021) 2021:9932218. doi: 10.1155/2021/9932218 34336123 PMC8289612

[56] Li JZ Li YR . Cardiovascular protection by metformin: latest advances in basic and clinical research. Cardiology. (2023) 148:374–84. doi: 10.1159/000531432 37307806

